# Multiple Faces of the Glioblastoma Microenvironment

**DOI:** 10.3390/ijms23020595

**Published:** 2022-01-06

**Authors:** Alina Simona Șovrea, Bianca Boșca, Carmen Stanca Melincovici, Anne-Marie Constantin, Andreea Crintea, Mariana Mărginean, Eleonora Dronca, Mihaela Elena Jianu, Rada Suflețel, Diana Gonciar, Maria Bungărdean, Carmen-Bianca Crivii

**Affiliations:** 1 Histology Discipline, Morphological Sciences Department, Iuliu Hatieganu University of Medicine and Pharmacy, 400000 Cluj-Napoca, Romania; simona.sovrea@umfcluj.ro (A.S.Ș.); bianca.bosca@umfcluj.ro (B.B.); carmen.melincovici@umfcluj.ro (C.S.M.); annemarie.chindris@umfcluj.ro (A.-M.C.); mariana.marginean@umfcluj.ro (M.M.); me05doc@yahoo.com (M.E.J.); sufletel_rada@yahoo.com (R.S.); 2 Medical Biochemistry Discipline, Molecular Sciences Department, Iuliu Hatieganu University of Medicine and Pharmacy, 400000 Cluj-Napoca, Romania; crintea.andreea@umfcluj.ro; 3 Medical Genetics Discipline, Molecular Sciences Department, Iuliu Hatieganu University of Medicine and Pharmacy, 400000 Cluj-Napoca, Romania; 4 Pathological Anatomy Discipline, Morphological Sciences Department, Iuliu Hatieganu University of Medicine and Pharmacy, 400000 Cluj-Napoca, Romania; dianagonciar@gmail.com (D.G.); maria.bungardean@yahoo.com (M.B.); 5 Anatomy and Embryology Discipline, Morphological Sciences Department, Iuliu Hatieganu University of Medicine and Pharmacy, 400000 Cluj-Napoca, Romania

**Keywords:** glioblastoma, microenvironment, intercellular communication

## Abstract

The tumor microenvironment is a highly dynamic accumulation of resident and infiltrating tumor cells, responsible for growth and invasion. The authors focused on the leading-edge concepts regarding the glioblastoma microenvironment. Due to the fact that the modern trend in the research and treatment of glioblastoma is represented by multiple approaches that target not only the primary tumor but also the neighboring tissue, the study of the microenvironment in the peritumoral tissue is an appealing direction for current and future therapies.

## 1. Introduction

Glioblastoma (GBM) is the most aggressive primary brain tumor in adults and accounts for approximately 60–70% of malignant astrocytoma. From a histological point of view, it is characterized by pathognomonic features, such as increased cellularity, mitotic activity, microvascular proliferation, and necrosis [1,2,3]. The tumor has a high infiltrative capacity (the extension of neoplastic astrocytes to the other cerebral hemisphere is fast via white matter tracts), it is highly heterogenic (tumor cells possess different molecular characteristics and dissimilar performances) and presents multiple phenotypes that quickly change, depending on divergent stimuli. These attributes, taken together, have a negative impact on treatment [1,2,3]. Therefore, GBM is considered a challenging tumor to treat. Under standard therapy (surgical resection, chemo-, and radiotherapy), the survival rate is about 15 months, mostly due to aggressive recurrences in the proximity of the original site [3].

Increasing evidence suggests that the tumor microenvironment (TME), a sophisticated aura that surrounds the neoplasm, represents a critical element impairing the success of anti-tumor therapy. As well as cancer cells, the TME contains several different non-cancerous cells (e.g., pericytes, fibroblasts, and endothelial and immune cells), many of which have local prevalence [4].

The intracellular and extracellular nanovesicles from tumor cells contain biomolecules (DNA, RNA, proteins, and lipids) that are captured by the resident non-neoplastic cells, subsequently triggering somatic and epigenetic signaling and fostering tumor progression [5].

Multiple molecular mechanisms expressed by these genetic (e.g., epidermal growth factor receptor (EGFF) overexpression, CDKN2A-p16 deletion, IDH1/2, and PTEN mutations) and epigenetic factors (e.g., non-coding RNA regulation, DNA methylation, histone modification, and chromatin remodeling) support very active crosstalk between the constituents of the TME, resulting in uncontrolled tumor proliferation, angiogenesis, and invasion [3,5].

**Figure 1:**
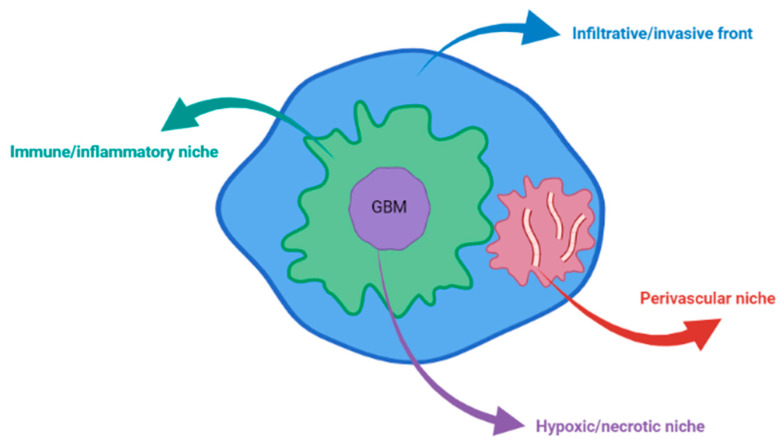
The four niches of TME in GBM.

Four major microenvironments have been described in GBMs, which constitute the GBM landscape and promote tumor cell reprogramming: the immune/inflammatory niche, the hypoxic/necrotic niche (in the tumoral core), the perivascular niche, and the infiltrative/invasive front (at the tumor edges) [6,7,8] (Figure 1). Inside these microenvironments, the cellular, biochemical, and biomechanical sectors modulate the TME [9].

The different types of cells that heavily infiltrate the GBM microenvironment (neurons, glial cells, glioma stem cells (GSCs), monocytes, macrophages, and lymphocytes) are activated by various signals, derived either from tumor cells or from their intense communication. Once activated, these cells generate an extensive degree of neuroinflammation through by an array of pro-inflammatory and cytotoxic cytokines, growth factors (e.g., tumor necrosis factor-alpha (TNFα), hepatocyte growth factor, epidermal growth factor (EGF), transforming growth factor-beta (TGFβ), and glial cell-derived neurotrophic factor), pro-inflammatory transcription factors (e.g., nuclear factor kappa beta (NF-κB) or signal transducer and activator of transcription 3 (STAT3)), matrix metalloproteinases (MMP-2 and 9), hydrolytic enzymes, bioactive lipids, reactive oxygen species (ROS), and nitric oxide (NO). This range of mediators manipulate, either by activation or by suppression, numerous signaling pathways, hijacking their primary role in tissue repair and finally promoting low anti-tumoral immunity, cancer cell survival, angiogenesis, and invasion [1,10,11].

Tumor growth is accompanied by hypoxia and abnormal vascular proliferation, activating inflammatory and immune cells (neutrophils, eosinophils, monocytes, dendritic cells, lymphocytes T and B, microglia, macrophages, and myeloid-derived suppressor cells, reactive astrocytes), and contributing to the chronic inflammatory status of TME and promoting continuous microglia activation [1,10,11].

The hypoxic status ensures a metabolic reprogramming of glioma cells and their precursors, efficiently utilizing energy and nutrients (amino acids, carbohydrates, lipids) for rapid tumor development and invasion in the surrounding healthy tissue. As a consequence of tumor growth in the peritumoral tissue, the metabolic changes trigger region-specific neuronal toxicity with neurodegeneration [5,12].

Hypoxic areas, created by the tumor’s high metabolic demands in the context of enlarged proliferation capacity, determine the prominent expression of vascular endothelial growth factor (VEGF) with immature vascular proliferation and necrosis (both typical features of GBM) [3].

Numerous mechanisms can be involved in developing tumor vasculature: angiogenesis (the proliferation of endothelial cells from the existing vessels), vessel co-option (incorporation of pre-existing vessels), intussusception (dilation or bifurcation of existing vessels), angiogenesis (recruitment of endothelial progenitor cells), vascular mimicry (tumor cells integrated into the endothelial lining), and differentiation of the stem-like cells into endothelial cells [13,14,15].

In addition to the control genes and epigenetic changes, all these different elements that form particular niches in GBM (inflammation, hypoxia, metabolic alterations, and vascular modifications) will be further detailed.

## 2. Genes and Epigenetic Factors

One of the first GBM classifications considered the origin of the tumors to classify them as primary and secondary neoplasms: primary or de novo GBMs (95% of cases) manifest rapidly, without any evidence of precursor lesions; secondary GBMs (5% of cases) usually develop from precursor lesions, such as low-grade diffuse astrocytoma and anaplastic astrocytoma [2,16].

In both cases, the tumor phenotype is the result of an interaction between its genotype and the surrounding microenvironment [16].

Molecular analyses have demonstrated that GBM can also be classified on the basis of molecular pathogenesis and “driver” lesions (i.e., molecular changes that initiate tumorigenesis and favor progression) [16,17]. Phillips classification of GBM based on transcription profiling provides the following subtypes of GBM: proneural with normal EGFR and PTEN expression, normal EGFR locus and no chromosomal alterations; proliferative with loss of PTEN expression, normal or amplified EGFR locus, and either gain of chromosome 7 or loss of chromosome 10; mesenchymal with loss of PTEN expression, normal or amplified EGFR locus [2,17].

Different types of molecular anomalies have been identified in gliomas and GBM, such as loss of CDKN2A, RB1, and TP53 tumor suppressor genes, in addition to mutations in the genes involved in these pathways or regulated by these tumor suppressor proteins [16,18,19,20,21,22]. For the secondary type of GBM, the most frequent genetic abnormality is the IDH mutation (60–80% of cases) which interferes with the metabolism, while the least frequent is PTEN loss (4%) which interferes with PIK3 signaling; For the primary type of GBM, the most frequent genetic abnormality is the TERT promoter mutation (60–80% of cases) which interferes with the telomere maintenance, while the least frequent is FGFR3-TACC3 fusion (3%) which interferes with RTK signaling [8,23,24,25,26,27,28].

Mutations in the IDH1, ATRX, and p53 genes are considered molecular hallmarks of diffuse and anaplastic astrocytomas (WHO Grades II and III) as well as secondary GBMs [16,20,23,24,25]; TP53 gene mutations occur in almost all cases of rare giant cell GBM [16,18].

In the majority of GBMs, the p53 (87% of GBM patients) and Rb (78% of GBM patients) pathways are disrupted, either by gene mutations or gene copy number variations [16,19,20]. Moreover, mutations in genes encoding the upstream regulators of Rb, or activation of the oncogenic pathways, such as those involving receptor tyrosine kinases (RTKs), may be found in the etiopathology of malignant gliomas [16,21].

In adult GBMs, there is a genomic amplification (40% of cases) in the EGFR gene, which is usually accompanied by internal deletions, as is the case for the variant EGFRvIII [16]. Although the changes leading to the EGFRvIII mutation are complex and heterogeneous, they are considered late events following EGFR amplification. EGFRvIII is found in 30–50% of cases in which EGFR amplification is present [2,16]. In 13% of adult GBMs, amplification of the platelet-derived growth factor receptor alpha gene (PDGFRA) is detected [2]. Similarly, activating deletions in PDG-FRA have been demonstrated in receptor-amplified GBMs [16].

Although much less frequent in GBMs, amplification of the MET proto-oncogene is also found [2,19,20]. Activation of genetic mutations can occur simultaneously in multiple RTKs in the same individual, resulting in cellular subpopulations with the mutations [21,22].

The majority of GBMs exhibit activation of the extended PI3K-AKT-mTOR and RAS-MAPK signaling pathways; therefore, these are considered as common oncogenic alterations. Mutations in these pathways include mutations in the genes encoding either the catalytic (PIK3CA) or regulatory (PIK3R1) domains of PI3K, which, in turn, induce the activity of these enzymes (in 15% of adult GBMs), as well as deletions and/or silencing mutations in PTEN, the primary negative regulator of the PI3K-AKT signaling pathway (in 30% of cases). Epigenetic and miRNA-based regulation of PTEN has also been described in diffuse gliomas, although they are more common in WHO Grade II and III gliomas (50–60%) [2,16,18,19,20].

Recently, neurofibromin 1 (NF1) somatic gene mutations or deletions (thought to be the cause of neurofibromatosis Type 1) have been identified in 15–18% of primary GBMs [2,16,20,21].

Mutations in Codon 132 of isocitrate dehydrogenase I (IDH1) and, less commonly, in Codon 172 are frequent in WHO Grade II and III diffuse gliomas (70–90%) and secondary GBMs (85%) but are rarely found in patients with what have traditionally been referred to as primary GBMs (5%) [16]. IDH1/2 mutations are generally found to positively correlate with other genetic abnormalities that are common to diffuse gliomas such as TP53 and ATRX mutations in astrocytomas and 1p/19q co-deletion in oligodendroglial tumors, while they display an inverse correlation with EGFR gene amplification and monosomy of chromosome 10, alterations that more commonly occur in primary GBMs [2,16].

More molecular abnormalities that appear to have an influence over the evolution and prognosis of GBMs have been identified in recent years [20].

DNA methylation is a mechanism of transcription regulation of oncogenes and tumor suppressor genes frequently identified in cancers. Methylation of gene promoters blocks transcription or produces structural chromatin changes via methyl-binding proteins [21]. Similar variations have been described in a subset of GBMs. For instance, IDH mutant gliomas show hypermethylation of CpG islands, known as the CpG island methylator phenotype (CIMP). These changes are presumably induced by the IDH mutations and, in turn, promote a less differentiated or stem cell-like state that is susceptible to other genetic changes, such as TP53 mutations or loss of chromosome 1p and 19q [20,21].

O6-methylguanine-DNA methyltransferase (MGMT) is a DNA repair enzyme that can reverse the effects of temozolomide (TMZ), the drug used as standard chemotherapy in GBM [20,21]. High MGMT activity levels can cause resistance to chemotherapy. Approximately 40% of GBMs might have this epigenetic change in the MGMT gene promoter, which leads to decreased MGMT activity and enhanced sensitivity to standard chemotherapy [21].

Telomerase reverse transcriptase is the catalytic component of telomerase that ensures the correct replication of telomeres; telomeres become shorter with every cell division, which is in direct relation to the lifespan of all cells, except cancer cells. In tumors, telomerase is abnormally activated, promoting the increased proliferation and lifespan of cells [21]. The most frequent mechanisms of telomerase activation are mutations of the TERT gene promoter, which are among the three most important genetic alterations in cancers, together with KRAS and TP53 mutations [24]. These mutations can be found in numerous cancers, such as melanoma, non-small cell lung cancer, bladder cancer, hepatocellular carcinomas, and GBMs [24,29,30,31]. TERT promoter mutations are found in approximately 80% of IDH wild-type GBM, as well as in the majority of IDH mutants, 1p/19q co-deleted oligodendrogliomas [21,22]. TERT promoter mutations in GBMs are associated with a worse prognosis than IDH wild-type GBMs [21,29].

Mutation of the histone H3 is seen in H3 K27M-mutant diffuse midline glioma, which is a subset of high-grade glioma [32]. These tumors are generally found in the pons, thalamus, and spinal cord in both adults and children, and are generally associated with a poor prognosis [33]. The histone mutation is mutually exclusive with IDH mutations but can co-occur with mutations in the receptor tyrosine kinase/Ras/phosphatidylinositol-3 kinase pathways [34]. The histone-mutated tumors often occur earlier in adults than in those of the median age for GBM [35]. In children, H3K27 tumors usually have a worse prognosis independent of the anatomical location.

In summary, many genetic alterations have been discovered that have both clinical and prognostic relevance. The progress in molecular classification of GBMs is extremely useful both for the therapeutical options and also for the prospect of developing more effective individualized and targeted therapy that will improve patient outcomes and survival.

## 3. Neuroinflammation: Cytokines and Oxidative Stress

Various pathologic processes causing varying degrees of neuroinflammation can disrupt the stable equilibrium of the neuroimmune system’s responses. Generally, cells in the CNS, along with peripherally derived cells, adapt dynamically, appropriately responding to the damage by releasing specific anti-inflammatory factors [36]. The neuroimmune axis responsible for the adequate response to homeostatic disturbances is kept intact by this multifactorial interaction, and potentially devastating neurotoxicity is minimized. However, disorders of the delicate mechanisms underlying this balance can lead to tumorigenesis [1].

Chronic inflammation promotes tumoral transformation and molecular changes by extrinsic and intrinsic mechanisms [1]. Intrinsically, carcinogenesis-related genetic actions trigger inflammation-associated programs that lead to the occurrence of an inflammatory microenvironment. Extrinsically, chronic inflammation stimulates tumor proliferation [1].

Usually, inflammatory mediators’ principal function is to clear out undesirable cells and encourage fibrous tissue formation to replenish the wounded area. Nonetheless, the inflammatory cells will react differently in the presence of chronic damage, secreting larger levels of immune inhibitory cytokines and other immunosuppressants [1]. Similarly, cytotoxic T cells and NK cells that infiltrate tumors produce inflammatory mediators, rather than having a tumoricidal action [37]. As a result, the inflammatory microenvironment generates a dysregulated immune response and supports tumor growth progression and invasiveness.

A large number of studies have shown the critical role of inflammation in the pathogenesis, behavior, and evolution of gliomas [38,39].

It is currently proven that the immune system can recognize GBM tumors and that these tumors are therefore susceptible to immune-mediated attacks [40]. Abnormal inflammatory processes are able to disorganize immune surveillance by generating an improper immunosuppressive TME. Consequently, they are highly responsible for the complicacy and harmfulness of GBM [41].

Chronic inflammation in the TME coordinates the interplay between resident non-tumoral astrocytes and tumoral ones, facilitating GBM invasion in healthy brain tissue. Two subtypes of astrocytes (A1 and A2) have been identified in the microenvironmental niche of GBM and initiate the inflammatory process [42,43]. Subtype A1 emerges post-lesion, is stimulated by pro-inflammatory factors to become reactive and has neurotoxic behavior. Subtype A2 maintains neuron connectivity and maintains neuronal integrity during hypoxia [44].

Chronic inflammation is also considered an inducer of the first genetic mutations that confer malignancy. If abnormal inflammation is a characteristic of cancers, it is also a driver of the malignant transformation of low-grade glioma. Due to aberrant signals in the genes involved in astrocyte development, astrocytes become tumorigenic [45]. FGF-2 is responsible for the neoplastic transformation of glioma cells and angiogenesis via Ras/Raf/ERK signaling [46].

**Figure 2:**
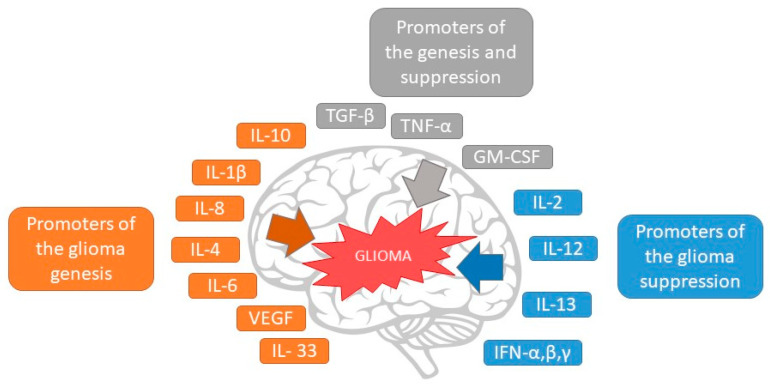
The effect of cytokines in glioma growth and suppression.

The inflammatory TME induces the liberation of numerous chemokines, cytokines, and growth factors that activate many signaling pathways (Figure 2). These signals allow tumoral cells to preserve constant growth, escape apoptosis, and attain metastatic ability [1,47].

Current research has revealed the molecular mechanism of inflammation that facilitates tumor development, in which cytokines represent the primary triggers. Cytokines are directly involved in various oncogenic processes, such as the interaction between tumoral cells and TME cells, the enrollment of inflammatory cells in the TME, cellular segregation, migration, invasion, and escaping immune mechanisms [48]. In GBM, a wide range of cytokines are present that play a crucial role in several pathways of tumor evolution [43]. The central cytokines involved are interleukins (IL) 1β, 4, 6, 8, 10, and 33, and several growth factors, such as TNF-α, macrophage migration inhibitory factor (MIF), and TGF-β [43]. These cytokines are expressed in an altered manner; IL-1β, TNF-α, IL6, IL10, IFN-γ, and CX3CL1 are overexpressed and are correlated with the onset of pain, as it was discovered that they are linked to tumor progression and aggressiveness [43].

Cytokines are a group of small molecules, glycoproteins, and polypeptides, that are secreted by specific cells of the immune system, and soluble mediators liberated by several cells in response to pathogens or tissue injury [1]. Normally, to sustain homeostasis, they assure the elimination of every unnecessary element from the nervous tissue and are critical as cellular signals in inflammation [1].

Thus, conditional on context-specific circumstances, cytokines might equally intervene in favor of pro-inflammatory or anti-inflammatory processes [1]. Finally, there are two types of cytokines: the pro-inflammatory ones (e.g., IL-6, 8, 1β, MIF, TNF-α) and the anti-inflammatory ones (e.g., IL-4,10 and TGF-β) [1].

C-reactive protein (CRP), IL-6, and TNF-α are circulating inflammatory markers and they are increased in the serum of GBM patients compared with healthy subjects. Thus, before initiating treatment, high levels of IL-6, IL-8, IL-17, IL-33, TNF-α, TGF-β, and CRP in the circulation are strongly associated with increased glioma stem cell activity that potentially leads to glioma progression and greater invasiveness [49]. Moreover, some research considered the increased levels of IL-6 and CRP as negative clinical predictors of evolution in patients with GBM [49]. The pro-inflammatory cytokines (such as TNF-α, MIF, IL-1β, and IL-6) initiate the inflammatory cascade [1].

TNF-α promotes T-cell growth and induces dendritic cell maturation in physiological conditions [50]. TNF-α is overexpressed in GBM, and its expression is associated with GBM tumor grade [50]. In the GBM TME, TNF-α secretion stimulates tumor development and angiogenesis [51]. TNF-α allows glioma cells to evade the immune response and to destructively expand in the inflammatory microenvironment by increasing both the expression of the major histocompatibility complex class (MHC-I) and its transcriptional activation (in parallel with amplified hypoxia-inducible factor 1-alpha (HIF-1α), ΝF-κΒ, and β-catenin actions) [52].

MIF is overexpressed in brain tumor-initiating cells [53]. Studies have found that MIF regulates p53, inducing cell proliferation and apoptosis in nervous tumor cells. Moreover, MIF may stimulate the liberation of the principal vascular triggers throughout the MAPK, CD44, and CD74 signaling pathways [54]. An intense association involving VEGF expressivity and MIF has been revealed in human GBMs [55].

IL-1β is a pro-inflammatory cytokine, mainly synthesized in astrocytoma and various brain tumors. It is responsible for tumor progression and metastasis and it is a crucial mediator in the proliferation of “reactive astrocytes” [56]. It seems that IL-1β is involved in the activation of NF-κB (via IκB kinase phosphorylation) and its subsequent alteration [57]. Research provided by Paugh et al. revealed that IL-1β enhanced tumor cell survival and invasiveness by stimulating Sphingosine kinase-1 upregulation [57]. IL-1β is highly expressed in the glioma microenvironment and, together with TGF-β, are responsible for the neurospheres induction, that increased expression of the stemness factor genes [58].

IL-6 represents an essential pro-inflammatory cytokine produced by many different cells (e.g., neurons, microglia, astrocytes, and peripheral monocytes) [1]. Contrary to the fact that under normal conditions, IL-6 assures neuroprotection, neurogenesis, reactive astrogliosis, and B cell maturation [1], increased levels of circulating IL-6 were established in oncogenic diseases (e.g., malignant melanoma, gastrointestinal tumors, and lung cancer), associated with tumor extent, stage, or development [1]. It was demonstrated that IL-6 has a pivotal role in brain tumor progression as well, initiating angiogenesis, cell proliferation, and resistance to apoptosis and facilitating cancer growth [59]. A significant association was demonstrated between IL-6 mRNA levels and the grade of glioma malignancy [60]. Li et al. (2010) revealed the potential of IL-6 to facilitate the GBM cells’ invasiveness [61]. In GBM (as detected by IHC), tumor cells themselves and peritumoral immune cells can release IL-6, increasing its circulating levels and suggesting that IL-6 may participate in glioma evolution in autocrine or paracrine ways [62,63]. In the peripheral monocytes of glioma patients, IL-6 levels are greatly increased compared with control patients [1]. Tumor-associated macrophages (TAM) and microglia present in the TME secrete significant quantities of IL-6, thereby causing the proliferation and metastasis of malignant cells [64].

IL-8 is another pro-inflammatory cytokine that is the main stimulator of angiogenesis, and it is greatly increased in most brain tumors [65]. It seems that the activation of NF-κB is responsible for the aberrant expressivity of IL-8 in GBM [66]. A study conducted by Carlsson et al. on subjects diagnosed with GBM designated IL-8 and VEGF as solid angiogenic elements [67]; moreover, the level of IL-8 is enhanced in high-grade gliomas such as GBM [68]. IL-8 also has a crucial role in GBM’s invasiveness: according to several studies, the invasive cellular capacity of GBM was greatly diminished upon the downregulation of IL-8 by short interfering (siRNA) [66,69].

IL-33 was recently demonstrated to be a pro-inflammatory cytokine; it initiates tumor progression, reduces survival, and increases resistance to immunotherapy in the GBM TME [70].

IL-4 is the most characterized anti-inflammatory cytokine in brain tumors [1]. IL-4 has a vital role in the immune evasion mechanisms of the GBM TME [1]. It is also responsible for the suppression of microglial synthesis of pro-inflammatory cytokines [1].

IL-10 is a significant anti-inflammatory and immunosuppressive cytokine produced in the brain cancer microenvironment [38]. It seems that IL-10 is greatly expressed in GBM [1]. A possible association between IL-10 mRNA and the tumor grade has been demonstrated [1]. IL-10 suppresses the multiplication of T lymphocytes, downregulates MHC Class II, and promotes the development of tumor evolution [1,38].

TGF-β is the central anti-inflammatory cytokine in brain tumors and is an immunosuppressive molecule [71]. The TGF-β family plays the leading role in adjusting the activity of tumor brain stem cells. TGF-β, especially TGF-β2, the primary isoform, is increased in GBM and stimulates the proliferation of cancer cells [1]. Moreover, TGF-β induces GBM cell movement and angiogenesis [71,72]. As a component of the Th3 cell’s response, TGF-β promotes the immunosuppression of T-cells [73].

Oxidative stress, combined with inflammation and chromosomal instability, creates a vicious cycle that stimulates brain oncogenesis [1]. Similar to other factors (e.g., cytokines, chemokines), many ROS and nitrogen species (RONS) are secreted by inflammatory cells. These factors stimulate oxidative stress in cells, leading to an altered DNA mismatch repair system (MMR), nucleotide excision repair, base excision repair, and MGMT; this will amplify DNA destruction, damage cell cycle barriers, and dysregulate the homologous recombination (HR) pathway [74]. DNA damage causes mutations. In GBMs, the main mutagenic DNA oxidation damage effect is 8-oxide [75]. It seems that enhanced 8-oxide in GBM increases histone γH2AX phosphorylation and promotes the DNA damage response that stimulates p53 [76].

Oxidative stress-induced MMR silencing frequently leads to microsatellite instability. RONS-mediated DNA damage associated with defective cell cycle checkpoints and HR causes genetic instability [77]. Genetic instability leads to upregulation of oncogenes and downregulation of tumor suppressor genes, which initiate glioma oncogenesis [77]. After activation, certain oncogene factors stimulate transcription factors such as NF-κB, STAT3, and HIF-1α, which ultimately secrete in favor of inflammatory chemokines and cytokines [1,78].

Cytokines and oxidative stress represent an encouraging therapeutic option in GBM treatment regarding their crucial implication in glioma development and progression.

The use of multiple inflammatory markers to predict patient outcomes was demonstrated to be a feasible solution. The recognition of these glioma indicators has a number of advantages: early treatment, monitoring of therapeutic effects, and an increase in the lifespan of patients.

## 4. Hypoxia

The brain has a high rate of metabolism and utilizes approximately 20% of the body’s oxygen consumption. In normal conditions, oxygen consumption is approximately 3.5 mL per 100 g of brain tissue per minute. This consumption is constant during both wakefulness and sleep [79].

In malignant conditions, the cancers are characterized by low oxygen levels, especially solid tumors that grow rapidly. Hypoxia represents a state in which oxygen deficiency in the tissues drives inadequate cell homeostasis. In the tumor microenvironment, chaotic cell proliferation leads to a need for oxygen far above the level that the existing blood vessels can provide.

Glioblastoma, a highly proliferative and heterogenic tumor, is characterized by a hypoxic microenvironment. In 2002, the oxygen levels were recorded in the peritumoral and intratumoral regions of glioblastoma: the results identified less oxygen in the intratumoral than in the peritumoral tissue: 1.25% vs. 2.5% O_2_ [80]. An oxygen gradient is created that influences the subsequent evolution of the tumor.

Clinical-pathological data can provide proof of oxygen restriction in the GBM. The pathological consequences of hypoxia consist of absent or defective blood flow detected by MRI [81,82] and the presence of multiple hypoxic regions with invasion into the periphery of the growing tumor, as noted in microscopic analyses [83].

Hypoxia, as a promoter of tumor growth and therapeutic resistance, activates various molecular mechanisms of glioblastoma evolution. HIF transcription factors (HIFs), heterodimeric complexes, represent the chief regulators of the adaptative response in hypoxia. HIFs constitute O_2_-regulated α subunits (HIF-1α, HIF-2α, and HIF-3α) and a constitutively expressed β subunit, HIF-1β (an aryl hydrocarbon receptor nuclear translocator (ARNT)) [84,85].

HIF-1α has a short half-life under physiological conditions, where it is rapidly degraded through the ubiquitin-proteasome pathway [86]. It becomes stable in hypoxic conditions, in which the β subunit transforms it into a functional transcription factor. By translocation into the nucleus, complete HIF-1 activates the expression of downstream genes as a response to hypoxia [87].

Related to the specific tumor microenvironments, HIF-1α (ubiquitously expressed) and HIF-2α (selectively expressed in distinct cell populations) play different roles in tumorigenesis [84], but they are considered to be the main regulators of the hypoxic response. On the other hand, variants of HIF-3α are under the control of distinct hypoxic mechanisms and a few variants regulate the functions of HIF-1α and HIF-2α [88].

However, hypoxia, as a response to changes in oxygen availability, acts as a trigger in cellular adaptation, most of the reactions being mediated by HIF, whose stability is altered under hypoxic conditions.

### 4.1. Genetic Alterations That Increase HIF Proteins

**Figure 3:**
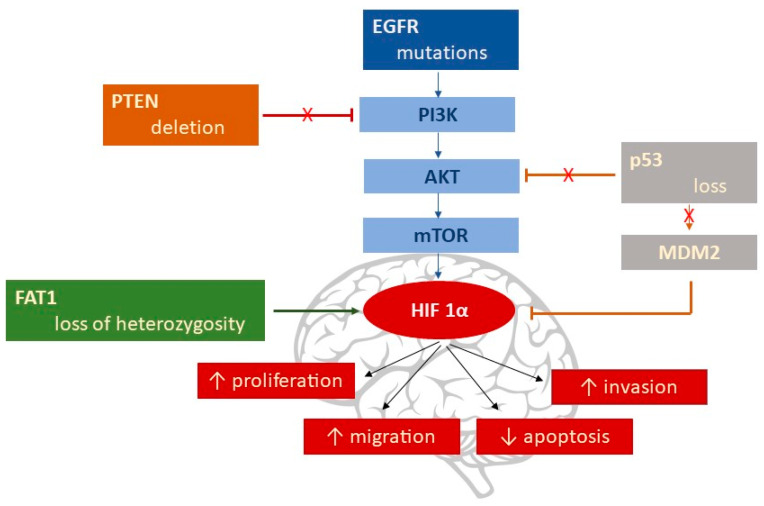
Genetic alterations that increase HIF-1α.

HIF regulation, in hypoxic conditions, is coordinated by many molecular pathways that influence the stability of HIF proteins or their capacity to bind to essential cofactors of transcriptional activity. The genetic factors responsible for HIF’s activation and further exacerbation in the hypoxic responses, are as follows (Figure 3):

- EGFR gene mutation by deletion of Exons 2–7 [89], followed by activation of the PI3K/AKT/mTOR pathway, with the subsequent upregulation of HIF-1α [90].
- PTEN (phosphatase and tensin homolog) deletions are found in 20–40% of GBM. PTEN is the main inhibitor of the PI3K/AKT/mTOR signaling pathway [91]; the result is an increase in HIF-1α via the PI3K/AKT pathway.
- Loss of the p53 gene leads to HIF-1α stabilization via MDM2-mediated ubiquitination, which is responsible for HIF-1α degradation under normal conditions [92,93]; p53 can be influenced by a variety of signals, including hypoxia.
- FAT1 (FAT atypical Cadherin 1) is linked to HIF-1α’s fate, being an upstream regulator of HIF-1α expression [94]. Endogenous depletion of FAT1 under hypoxic conditions was accompanied by a decrease in HIF-1α expression, along with its downstream target genes (CA9, GLUT1, VEGFA, MCT4, HK2, BNIP3, and REDD1) [95]; the result was an important diminution in the GBM’s aggressiveness. Loss of FAT1 heterozygosity has been found in astrocytic tumors [96].

### 4.2. Maintenance of Glioma Stem-like Cells

Glioma stem-like cells (GSCs) are known to play a crucial role in the development, maintenance, and recurrence of glioblastoma. GSCs are related to glioblastoma’s resistance to therapy and are maintained by the hypoxic microenvironment. Single-cell RNA and genome sequencing analyses studies have revealed the heterogeneity of the cells in glioblastoma, with characteristics of hypoxia as well as stemness [97,98]. The impact of chromosomal instability contributes to the genetic and transcriptional heterogeneity among glioblastoma cancer cells [99].

The low-oxygen environment stabilizes HIFs, which, in turn, maintain the stem-like phenotype of certain cells [100]. HIF-1α is linked to the tumorigenic capacity and expansion of GSCs, especially in peri-hypoxic niches [101], but also has an important role in peri-vascular and peri-arteriolar niches [102]. The GSC markers (SOX2, OCT4, CD9, CD133, and nestin) indicate the differentiation of glioblastoma cells into GSCs [103,104]. HIF-1α and HIF-2α are present in the immunohistochemical staining of glioblastoma biopsies in these peri-hypoxic niches [101]. While HIF-1α has more general effects on GSCs’ survival, HIF-2α specifically upregulates the expression of SOX2, OCT4, and CD133, promoting the GSC phenotype [101,105].

### 4.3. Hypoxic Metabolic Adaptation

**Figure 4:**
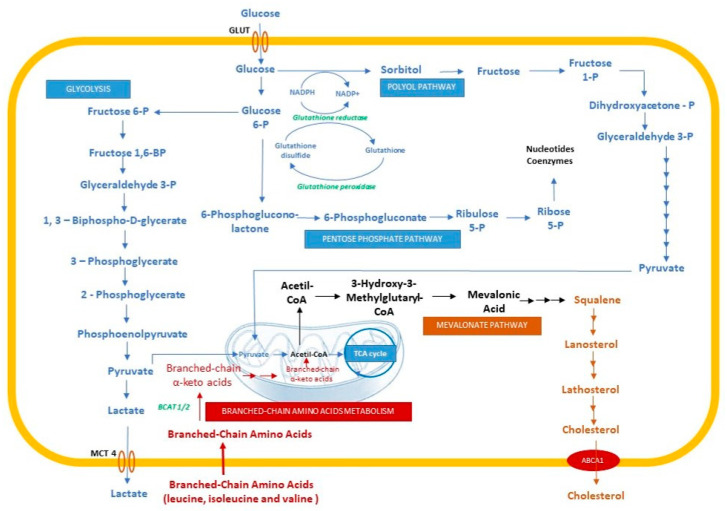
Metabolic changes in hypoxic cells. GLUT, glucose transporter; MCT4, monocarboxylate transporter 4; ABCA1, ATP-binding cassette transporter.

Glioblastoma is characterized by hypoxic stress that triggers metabolic adaptive responses. Prolonged exposure to hypoxia leads to significant metabolic changes, with a strong accumulation of metabolites in hypoxic cells (Figure 4).

Under normoxic conditions, cellular growth and division are processes that use energy as a result of metabolic reactions, consuming the nutrients from the surrounding environment. The high metabolic rate of the brain uses around 60% of the daily glucose intake [106]. Due to its inability to store glucose as glycogen, the brain depends upon a constant supply of glucose. At the same time, a high glucose level is linked to increased tumor invasion and the poor survival rate of patients [107,108].

Excessive intake of nutrients induces the implication of different systems to prevent individual aberrant proliferation. Cancer cells benefit from signaling pathways that activate the absorption and metabolism of nutrients to promote growth, survival, proliferation, and function [109,110].

The main process of cellular energy production is mitochondrial oxidative phosphorylation (OXPHOS), not aerobic glycolysis [111]. Despite the competition for glucose between tumor cells and stromal and immune cells, tumor cells take up increased amounts of glucose, which leads to increased glycolysis and lactic acid fermentation, with a decrease in OXPHOS. This is a feature of tumor metabolism known as the Warburg effect [110,112]. Gene expression analyses for GBM cells emphasize the increased levels of transcripts encoding proteins that are involved in glucose uptake (by approx. 11-fold) and glycolysis, with several intermediates [113]. In hypoxic cells, HIF-1α diminishes pyruvate flux into the tricarboxylic acid (TCA) cycle by pyruvate dehydrogenase kinase (PDK)-mediated inhibition of pyruvate dehydrogenase (PDH) [114,115]. Pyruvate accumulation is converted to lactate by LDHA (lactate dehydrogenase A) and then translocated into the extracellular space by monocarboxylate transporter 4 (MCT4) [116,117]. Data on gene expression showed that hypoxia induces a transient increase in PDK and MCT4, and analyses of metabolites showed a significant increase in lactate in hypoxic cells compared with normoxic glioblastoma cells [113]. Moreover, Bartrons et al. demonstrated that there is a metabolic reprogramming of tumoral cells by the cross-transfer of lactate from cancer-associated fibroblasts (whose metabolism takes place in hypoxic conditions where ROS, HIF-1α, and NF-κB induce glycolysis) to cancer cells where the lactate is transformed to pyruvate and consumed in the TCA cycle. The result is a high mitochondrial OXPHOS level and low glycolysis, associated with low apoptosis and high proliferation [117].

In hypoxic cells, glucose and glycolysis intermediates are not the main source of energy but they are the substrates for macromolecular synthesis [118], generating sufficient energy (ATP) for cellular reactions [119]. Hypoxia induces the levels of both sorbitol (by approx. threefold) and fructose (by up to >80-fold), indicating the profound activation of the alternative glucose metabolism route, the polyol pathway [113]. The polyol pathway appears to intensify cancer progression, demonstrating resistance to hypoxia.

Increased glucose flow to the polyol pathway alters the metabolic cytosolic pyridine nucleotides to provide an increased ratio of NADP^+^/NADPH, which sustains the pentose phosphate pathway (PPP), and NADH/NAD^+^, which may inhibit the transformation of glyceraldehyde-3-phosphate into 1,3-bisphosphoglycerate, promoting the process of transformation of glucose into diacylglycerol (DAG) or pentoses through PPP [120].

In addition, in hypoxic conditions, an increased ratio of NADP^+^/NADPH and NADH/NAD^+^ compromise the reduction of glutathione, leading to oxidative stress and the generation of ROS. Oxidative stress can induce damage to different molecules, such as proteins, lipids, and DNA, leading to genomic instability [121]. In hypoxic conditions, the cell response involves the HIFs and endoplasmic reticulum (ER) stress responses [122] as an adaptative reaction achieved by the active upregulation of a set of genes that creates a microenvironment that is supportive of tumor progression [123].

Oncogenic signaling and glioblastoma have recently been linked to dependence on lipids rather than glucose as a primary substrate for energy production [124,125].

MR imaging studies in patient tumors reported that elevated lipid resonances are a characteristic of hypoxic tumor areas [126]. Lipoproteins are internalized by endocytosis by binding to lipoprotein receptors (very low-density lipoprotein receptor (VLDLR), low-density lipoprotein receptor (LDLR), low-density lipoprotein-related protein (LRP1)) and scavenger receptors (SR-B1) [127]. Increased rates of lipid synthesis occur through the increased expression of various lipogenic enzymes. There is ample evidence that increased lipid production is essential for cancer cell survival, while the expression of a central lipogenic enzyme, fatty acid synthetase (FAS), is strongly correlated with cancer progression [128].

In normal conditions, brain cholesterol, a major component of the cell membrane and a regulator of cell signaling, represents 20–25% of total body cholesterol [129]. Despite the incapacity of peripheral cholesterol to pass the blood–brain barrier (BBB), the necessary cholesterol is synthesized de novo by the astrocytes and transferred to the neurons within high-density lipoproteins containing apolipoprotein E (Apo-E) [129,130].

Hypoxic glioblastoma cells reveal the accumulation of squalene, lanosterol, and lathosterol, molecules that need oxygen for conversion into cholesterol [129]. Kambach et al. demonstrated that densely plated glioma cells increase the synthesis of cholesterol by enhancing oxygen consumption, glycolysis, and PPP [131]. Furthermore, the densely plated normal astrocytes downregulate the genes involved in the control of cholesterol synthesis, such as farnesyl diphosphate synthase, farnesyl-diphosphate farnesyl-transferase 1, and squalene epoxidase (FDPS, FDFT1, and SQLE) [132]. Villa et al. demonstrated that GBM needs cholesterol to survive [133], highlighting the effect of LXRs, a transcription factor, which increases the expression of the cholesterol efflux regulatory protein (CERP) (also known as the ABCA 1–ATP-binding cassette transporter), a molecule that facilitates the efflux of cholesterol. The LXRs agonists induce glioma cell death through the low level of cholesterol [133].

Amino acids are important fuels for promoting the growth and division of tumor cells [134]. Normally, cells use the essential amino acids for protein synthesis. These amino acids are not synthesized de novo by human cells; they are derived from the diet [135]. However, there are different types of cancer that use different sources to synthesize the essential and non-essential amino acids [136]. These biosynthesis pathways are under the control of oncogenic signaling and depend on the tumor’s origin [137,138]. Branched-chain amino acids (BCAA) are a group of three essential amino acids: leucine, isoleucine, and valine, which are essential nutrients for tumor growth and are used as a source of energy [139]. Cytosolic branched-chain aminotransferase 1 (BCAT1) and mitochondrial branched-chain aminotransferase 2 (BCAT2), metabolic enzymes of BCAA, appear to vary between cancer types, but BCAT1′d expression is correlated with increased aggression of growth and progression [139].

HIFs mediate the response of amino acids to hypoxia. Zhang et al. demonstrated the role of HIFs (HIF-1 and HIF-2) in the upregulation of L-type amino acid transporter 1 (LAT1) protein and BCAT1, a metabolic enzyme implicated in the transport and metabolism of BCAA [140]. There is evidence of HIF’s action on glutamate tagging from BCAA. Under hypoxic conditions, blocking HIF-1α and HIF-2α leads to decreased detection of glutamate from BCAAs, which provides functional evidence for HIF intervention in reprogramming BCAA metabolism [140].

### 4.4. Epithelial-Mesenchymal Transition

EMT is a process involved in tumor progression, invasion, and metastasis.

FAT1 is a major factor involved in various hypoxic mechanisms, including EMT. FAT1 expression positively correlates with the expression of hypoxia, EMT, and stemness in GBM tumors, while FAT1 knockdown decreases the expression of all these [141]. In a previous study, it was demonstrated that FAT1 upregulates HIF-1α’s expression and functions in hypoxic conditions, changing the invasive capacity [95]. Under severe hypoxia, FAT1 knockdown led to decreased expression and function of HIF-1α, along with decreased migration and invasion of GBM cells [95].

HIF-1α suppression reduces the expression of all EMT markers (Snail, LOX, N-cad, and Vimentin), as in FAT1 knockdown. At the same time, nestin, which is involved in GBM cell invasion, and SOX2, a marker of stem-like cells, are not reduced by HIF-1α knockdown but are downregulated by FAT1 knockdown [141,142]. SOX2 expression is primarily under the control of HIF-2α [143].

TIPE2 (tumor necrosis factor-α (TNF-α)-induced protein 8-like 2), a member of the TNF-α-induced protein 8 (TNFAIP8, TIPE) family, maintains immune homeostasis [142,144] and it was demonstrated to inhibit the proliferation and invasion of different tumor cells [145,146]. Overexpression of TIPE2 prevents hypoxia-induced expression of β-catenin, cyclin D1, and c-myc in human glioma cells, suggesting that TIPE2 overexpression inhibits hypoxia-induced activation of the Wnt/β-catenin pathway and EMT in glioma cells [147].

In different types of solid tumors, researchers have indicated the implications of HIF-1α in the regulation of EMT transcription factors, enzymes (e.g., lysyl oxidase (LOX)), MMPs (such as collagenase MMP1 and gelatinase MMP2), histone modifiers (e.g., histone lysine-specific demethylase 4B (KDM4B)), adhesion molecules (e.g., angiopoietin-like 4 (ANGPTL4), L1 cell adhesion molecule (L1CAM)), chemokine receptors 1 and 4 (CX3CR1, CXCR4), and miRNA targets to stimulate tumor progression and invasion [148,149].

Following the description of the processes in which hypoxia occurs, it can be stated that hypoxia represents a major concern for GBM patients due to its capacity to invade the healthy brain tissue via different mechanisms. Tumor invasion is a major obstacle to surgery, radiotherapy, and chemotherapy, but it is also the main cause of death in GBM patients. Understanding how hypoxia triggers the molecular systems of GBM to become invasive is crucial for developing new and more effective therapies against this overwhelming disease.

## 5. The Tumor Microenvironment and Vascular Modifications

GBM is one of the most highly vascularized solid tumors, with an intense vascular proliferation and hyperplasia of the endothelial cells (ECs) [150,151,152].

GBM neovascularization includes two major processes: vasculogenesis and angiogenesis. Vasculogenesis represents the formation of new (de novo) blood vessels, mainly after the recruitment and differentiation of bone marrow-derived cells, while angiogenesis or neoangiogenesis in the development of new blood vessels, is based on the proliferation and migration of preexisting ECs [150,153].

The newly formed tumoral vessels establish anarchic, abnormal vascular networks; they are dilated, tortuous, and highly permeable, with aberrant ECs, detached pericytes, and a thick basement membrane; subsequently, the BBB is compromised and parenchymal edema appears [150,151,152,154,155,156,157]. These blood vessels can also form aggregates of small dysfunctional vascular structures, similar to renal glomeruli, called glomeruloid microvascular proliferation (GMP), a common hallmark of GBM [155,156].

The new blood vessel formation is initiated by hypoxia and the secretion of HIF-1 in the TME. The intratumoral HIF-1 induction leads to the recruitment of bone marrow-derived cells (BMDC), which initiate the process of vasculogenesis [158].

There is low oxygen delivery to the cells and high oxygen consumption by hyperplastic cells [150,152,154]. As a consequence, pro-angiogenic factors are activated: VEGF, basic fibroblast growth factor (bFGF), acidic fibroblast growth factor (aFGF) [157], platelet-derived growth factor (PDGF-β), angiopoietin-1 (Ang-1), angiopoietin-2 (Ang-2), TGF-β, TNF-α [150,152,154,159], hepatocyte growth factor (HGFR/c-MET) [150,157,160], ephrines [150,152], IL8 [150,152], VE-cadherin [159]. These factors bind to tyrosine kinase transmembrane receptors (RTKs), activating signal-transducing pathways that lead to new vessel growth [157]. It is also worth mentioning that the inflow of BMDC can restore damaged vascularity by irradiation [158].

Due to the severe hypoxia, the pseudopalisades which surround the necrotic foci in GBM secrete proangiogenic factors. The pseudopalisades describe a group of actively migrating tumor cells from the central hypoxic area. Pseudopalisading cells are known to be hypoxic, as demonstrated by their dramatic upregulation of HIF-1 [81].

The microvascular hyperplasia provides new vessels and supports the tumor expansion associated with the evolution of the pseudopalisades. The microvasculature, in most cases, is directly adjacent to the pseudopalisades [161].

One of the pro-angiogenic factors is VEGF-A, also called VEGF, which is upregulated by HIF-1α. It is the most essential and potent stimulator of angiogenesis, a key factor used by tumors to switch to their angiogenic phenotypes [150,152,154]. In response to oxygen deprivation, VEGF is secreted by endothelial cells but also by tumor cells and TME cells, including macrophages and other BMDC [154,162].

Activation of VEGF-A RTKs, such as VEGFR-1 (Fms-like tyrosine kinase 1) and VEGFR-2 (kinase insert domain receptor in humans; fetal liver kinase 1 in mice), which are expressed in tumor and stromal peritumoral cells [154,162,163], plays a vital role in GBM angiogenesis from regions adjacent to the pseudopalisade with a high vascular density [81]. The receptor activation triggers the activation of signaling pathways involved in the proliferation and migration of endothelial cells, the inhibition of apoptosis, ECM degradation, and the development of chemoresistance [152,160,162,164]. VEGF-A can bind to both VEGFR-1 and VEGFR-2, but it has a 10-fold higher binding affinity for VEGFR-1 [154].

Placenta growth factor (PlGF) is also a growth factor of the VEGF family that activates VEGFR-1 and is highly expressed in high-grade gliomas [162].

Most studies revealed that VEGFR-2, which is greatly expressed in GBM, is the most active receptor in GBM angiogenesis, mediating the angiogenic, mitogenic, and permeability-enhancing effects of VEGF-A [157,159,162]. Moreover, VEGFR-2 can be activated not only by binding to VEGF-A but also by interacting with membrane-associated integrins, leading to destabilization of the intercellular junctions and thus increasing vascular permeability [152,154,159].

**Figure 5:**
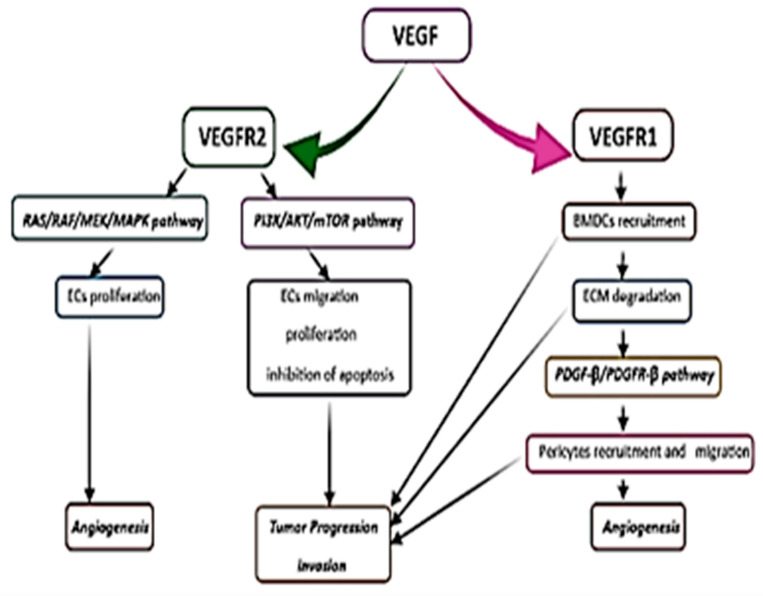
Representation of tyrosine kinase transmembrane receptors (RTKs) and signal-transducing pathways that lead to new vessel growth, tumor progression, and invasion. ECs, endothelial cells; BMDCs, bone marrow-derived cells.

Activation of VEGF RTKs leads to the downstream signaling of two major molecular signaling pathways: RAS/RAF/MEK/MAPK and phosphatidylinositol 3-kinase (PI3K)/protein kinase B or AKT/mammalian target of rapamycin (mTOR) [152,157,160]. If VEGF induces the proliferation of endothelial cells via activation of the MAPK pathway [152], the PI3K/AKT/mTOR pathway is involved in proliferation, migration, and cell survival, promoting GBM progression, so it is of central importance in VEGF signaling [152,157,160]. Several studies revealed that inhibition of the PI3K/AKT pathway might inhibit the growth and invasion of GBM (Figure 5) [160].

Another important mechanism involved in GBM angiogenesis, apart from the migration and proliferation of the ECs, is the recruitment of pericytes and bone marrow-derived cells (monocytes, macrophages, and hematopoietic stem cells) in the perivascular tumor niche due to the activation of VEGFR-1 [152,154,155,156]. As a result, inflammatory cytokines are released from the inflammatory cells (TNF-α, IL-1β, IL-6, IL-8, etc.) [152,154], promoting tumor invasion and progression. Some authors suggested that IL-8, mainly secreted by the macrophages in hypoxic/anoxic conditions, contributes to GBM angiogenesis and progression [152,156]. Its highest level is found within the pseudopalisades in the tumor resection margin. Because of its lower accumulation in the peritumoral tissue, IL-8 is associated with invasion and angiogenesis at the tumor border [165].

Vascular pericytes or the mural cells of blood vessels have an essential role in preserving the vascular integrity and maintenance of the BBB. Besides their contractile function, the pericytes may exhibit stem cell properties, playing an important role in new blood vessel formation [5,10]. VEGF ligands, binding to RTKs, promote pericyte detachment and vessel instability, forming a large, anarchic, and abnormal network of hyperpermeable blood vessels [157], known as glomeruloid microvascular proliferation (in a similar manner to kidney glomeruli) [152,156]. The PDGF-β/PDGFR-β signaling pathway is crucial in pericyte recruitment and new blood vessel formation [154].

The extracellular matrix degradation and remodeling via activation of MMPs, after stimulation of VEGFR-1, will activate the PDGF-β/PDGFR-β pathway and the migration of pericytes, thus promoting GBM angiogenesis and invasion [152,154,157,166,167]. MMP-2 and MMP-9 are greatly expressed in patients with high-grade gliomas [157,166,167].

The PDGF expression of endothelial cells and mural vascular cells correlates with GBM progression and angiogenic activity [152,154]. The PI3K/Akt, MAPK/ERK, and STAT3 signaling pathways play an important role in the angiogenic effects of PDGF [152].

VEGF secretion may determine the activation of other pro-angiogenic factors (Ang-1, Ang-2) involved in glioma vascularization [152]. VEGF stimulates the angiogenic effect of Ang-2, inducing proliferation, migration of the endothelial cells, and vasodilatation [152]. Ang-2 and its receptor, Tie-2, are not expressed in the normal brain’s vascular endothelial cells but they are present in the small blood vessels of GBM, suggesting their role in the early stages of angiogenesis [152,157]. In the absence of VEGF, Ang-2 acts as an antagonist of Ang-1 to have an anti-angiogenic effect.

Several authors have provided some information about a new mechanism involved in the formation of GBM vasculature, such as vasculogenic mimicry (VM) [151].

Vasculogenic mimicry represents a new type of tumor neovascularization, characterized as already presented, by a network of functional fluid-conducting vessel-like structures containing red blood cells (RBC) embedded into the tumoral matrix. These newly formed tubular structures have a PAS+ basal lamina and are lined by cells that derive from GSCs (CD133+GSCs), showing the properties of endothelial cells [151,153,156,159].

GSCs (CD133+GSCs) are located mostly in perivascular areas, particularly in the tumor niches of GBM, and are considered chemo-/radioresistant, and thus are responsible for tumor recurrence [151,168]. Under hypoxic conditions, activation of the PI3K/AKT signaling pathway stimulates VEGF expression in CD133+GSCs [151]. As presented before, CD133+GSCs may undergo mesenchymal differentiation, transdifferentiating into functional endothelial-like cells and smooth muscle-like cells (similar to vascular pericytes) [155,156,159], thus supporting the formation of new blood vessels. Therefore, a high proportion of the cells that line the newly formed tumoral vessels derive from tumoral cells CD133+GSCs [155]. The pathogenesis of VM in GBM is not well understood, but some authors have correlated VM with poor prognosis and the development of GBM resistance to anti-angiogenic therapies [151,153,157,159,168,169]. A series of recent studies have indicated that SU1498 and AZD2171, potent inhibitors of VEGFR-2, reduce VM formation in vitro and in vivo, as well as the aggressiveness of GBM [157].

Anti-angiogenic therapies in GBM, mainly silencing the VEGF/VEGFR-2 signaling pathway, are still under clinical investigation. Bevacizumab, which inhibits VEGF-A, represents the most widely used anti-angiogenic agent for GBM, being approved by the FDA for treating recurrent GBM [155]. However, this anti-angiogenic agent showed only reduced efficacy, partially (maybe) because of VM.

Some studies have suggested that understanding the mechanisms of VM will improve GBM therapy in the future, providing new targets for anti-angiogenic therapies.

## 6. Conclusions

The complicated picture of the GBM microenvironment influences the overall biology of GBM and increases its resistance to therapy. Further insights into the microenvironment in GBMs will probably facilitate an understanding of the molecular mechanisms that enable tumor initiation, development, and progression, and will lead to a novel design of therapeutic strategies to fight cancer in a personalized manner.

Knowing how each component of the TME interacts with the tumor and the rest of the TME would help target treatments to those patients who will benefit, thus reducing the current clinical burden of this cancer.

However, many critical questions regarding the roles of the microenvironment in GBM’s progression remain elusive. To answer these questions, a more profound knowledge of the crosstalk between the cancerous glioma cells and their microenvironment is needed. Finally, more research must be completed until a more comprehensive understanding of this complex environment is attained.

## Data Availability

Not applicable.
